# A Definition of Conditional Probability with Non-Stochastic Information

**DOI:** 10.3390/e20080572

**Published:** 2018-08-03

**Authors:** Pier Giovanni Bissiri, Stephen G. Walker

**Affiliations:** 1 School of Mathematics, Statistics and Physics, Herschel Building, Newcastle University, Newcastle upon Tyne NE1 7RU, UK; 2 Department of Mathematics, University of Texas at Austin, 2515 Speedway, Austin, TX 78712, USA

**Keywords:** conditional probability, loss function, non-stochastic information

## Abstract

The current definition of a conditional probability enables one to update probabilities only on the basis of stochastic information. This paper provides a definition for conditional probability with non-stochastic information. The definition is derived by a set of axioms, where the information is connected to the outcome of interest via a loss function. An illustration is presented.

## 1. Introduction

The theory of conditional probability is a well-established mathematical theory that provides a procedure to update probabilities by taking new information into account. The new work in this paper is motivated by the fact that such a procedure is available only if the information which is used to update the probability concerns stochastic events, that is, events to which a probability is assigned. In other words, such information needs to be already included in the probability model. The statistical implications are most pertinent to Bayesian inference where non-stochastic information, obtained by experts for example, is often employed.

To set the scene, Ω denotes the set of possible outcomes of an experiment, which we assume is finite, p(ω) is a probability mass function on Ω, and *I* is an arbitrary piece of information about the unknown outcome of the experiment, i.e., information for which one would consider revising p(ω). We argue that there *must* be a formal way to derive the updated probability mass function, which we write as $p_{I}\left(\omega \right)$. For example, Ω might represent a horse race and a bookmaker has set *p*. It starts to rain, so I= “it is raining”, and the bookmaker revises *p* to $p_{I}$. One imagines that the bookmaker does so from experience and uses no formal rule to implement the change. This lack of a formal rule then questions the extent of the relevance of subjective probability if one can appeal to no formal mechanism for revising belief distributions with arbitrary, yet relevant, information. Recall that the formal Bayes conditional probability rule requires the specification of p(I|ω) for all possible pieces of information *I* that one could receive–to us, at least, this is an impossible task.

It is impossible to set the probabilities p(I|ω) *a priori* for all possible *I*, yet this is required for the validation of the mathematical implementation of the Bayes rule. However, setting a loss function l(ω,I) once *I* has been received, is, we argue, a totally viable task. This would establish the loss, on some scale, e.g., monetary, if the elementary event ω occurs once *I* has been received. Our formal updating rule relies on the construction of such l(ω,I).

*I* is a piece of information for which it is possible to construct a loss function l(·,I) on Ω that is valued into [0,∞] and is not identically infinite. So, l(ω,I) is the loss that the experimenter assigns to the outcome ω in Ω when holding the piece of information *I*. We look at an illustration involving such l(ω,I) later on in the paper. Once *I* has been received, the probability of updating is *P*, where $P\left(B\right)=\sum_{\omega \in B}p\left(\omega \right)$ for every B⊂Ω.

For example, if *I* is the information “event B⊂Ω has occurred” and

(1) $$l\left(\omega ,I\right)=\left\{\begin{array}{cc} \infty & \omega \notin B\\ 0 & \omega \in B \end{array}\right.$$

then our formal rule will coincide with the usual definition of conditional probability.

When the standard definition of conditional probability does not apply, e.g., *I* is not such a statement or *B* is not a subset of Ω, for example, an alternative definition based on a mathematical decision theoretic framework can be used. When information received is non-stochastic, but relevant to an outcome of interest, we cannot use a probability distribution and so we need an alternative way to connect the information *I* with the outcome of interest ω. We do so using loss functions and a set of axioms.

This paper provides a definition for conditional probability on the basis of *I*. Reference [1] addressed this issue considering the minimization of a cumulative loss function which involved *g*-divergences. In this paper, instead, the definition of conditional probability is solely based on a set of axioms. This idea was developed by reference [2] who defined a framework for general Bayesian inference and discussed its application to important statistical problems. The present paper also addresses the issue of calibrating the conditional probability with non-stochastic information.

### 1.1. Relationship to the Literature

In the literature, alternative definitions of conditional probability, such as the Jeffrey’s Rule of conditioning, are given where new information is not put in terms of the occurrence of an event included in the model. These definitions rely on the assumption that information can be given in the form of a constraint on the probability. Constraints considered are of the type

(2) $$\sum_{\omega \in \Omega }g\left(\omega \right)p_{I}\left(\omega \right)>0,$$

where *g* is a measurable real function on Ω, and $p_{I}$ is the updated probability function. The idea is to minimize the Kullback–Leibler divergence subject to the constraint (2), which represents the information *I*. This problem can be solved using Lagrange multipliers.

For more detail about conditionalization based upon constraints on the conditional distribution, see references [3,4,5,6,7,8]. Our approach is different as we can deal with more types of information. On the other hand, our definition can encompass potentially arbitrary information about the outcome; all we need is to construct a loss function l(ω,I) for each ω in Ω once *I* has been received.

### 1.2. Motivation

The need for a definition of conditional probability outside of the usual set-up is the avoidance of paradoxes where one knows event *B* has occured yet is not a subset of Ω. Paradoxes arise when *B* is not deemed to be part of the stochastic model yet is subsequently forced to be so.

Such difficulties arise in different puzzles, such as, for instance, Freund’s puzzle of the two aces, introduced by reference [9]. For other puzzles about conditional probability, see, for instance, reference Gardner [10].

These puzzles have been widely used to discuss the concept of conditional probability. According to reference [11], such a concept is justifiable only on the basis of “a set of rules that tell, at each step, what can happen next”, which he calls “protocol”. For conditioning on an event *B*, he asserts that “we are assuming *B* is in your probability model, i.e., in the field of events to which you assign probabilities. So it is implicit in the principle of total evidence that your probability model should include a model for what you learn”. On the other hand, Hutchison [12,13] emphasizes that the updating process needs to take into account the circumstances under which the truth of *I* was conveyed. Also, Bar–Hillel and Falk [14] claims that knowing how knowledge was obtained is “a crucial ingredient to select the appropriate model”. These authors present different views about the concept of conditionalization, but all agree on the fact that there would not be a problem if it was known how the information *I* had become available and therefore, one could build a model including *I*.

The concept of conditional probability distributions is certainly appropriate as a procedure to update probabilities on the basis of any new information that has already been included in the probability model. However, it can be difficult to construct a model that considers all possible relevant information that could become available in the future. Therefore, a problem arises when one obtains some new and possibly unexpected information and wants to use it to update a probability distribution. Indeed, it does not seem appropriate to assess the probability of something which has been already observed. Our basic assumption is that the information *I* can be connected to the outcome of interest via a loss function l(ω,I). In this way, it is possible to update the probability *P*, even if *I* is some new unexpected information that was not included in the probabilistic framework.

Clearly, we are dealing with information about outcomes of interest which take many forms. It is well-known that Bayesian inference can rely on expert opinions which often take the form of non-stochastic information. In particular, our framework allows coherent updates of probabilities involving such information, and hence, the practical relevance of our framework to Bayesian inference.

## 2. Results

This section reports the current definition of conditional probability and presents and motivates our definition for conditional probability with non-stochastic information.

### 2.1. The New Definition

If p(ω) represents prior beliefs about ω, then we argue that a valid and coherent update of p(·) on the basis of *I* is the posterior, $p_{I}\left(\cdot \right)$, where

(3) $$p_{I}\left(\omega \right)=\frac{\exp\{-\lambda l(\omega ,I)\}p(\omega )}{\sum_{\omega^{\prime }\in \Omega }\exp\left\{-\lambda l\left(\omega^{\prime },I\right)\right\}p\left(\omega^{\prime }\right)},$$

and λ is a positive constant.

Looking at this form, it seems that we are proposing the equivalent of a Bayes rule with

(4) p(I|ω)=exp{−λl(ω,I)}.

This is correct only if the piece of information *I* is an element of a known space of possible outcomes. Indeed, only in such a case would it be possible to assess a probability distribution in such a space.

In our proposal, a much less complex framework is considered, since it is required to just set the loss l(ω,I) once *I* has been received. Our axioms which lead to (3) do not regard (4) as the probability.

We shall now go into the details of how some natural assumptions imply (3). The following axioms are considered:

1. The posterior form is given by
  $$p_{I}\left(\cdot \right)=\psi \left(l\left(\cdot ,I\right),p\left(\cdot \right)\right),$$
  for some function (ψ). Since l(·,I) and p(·) are all that are available and set, it is clear that the update must solely depend on these functions. We are, hence, asking for the unique ψ which provides the update for all Ω, i.e., Ω invariant. This is a reasonable requirement since how one updates should not depend on Ω.
2. If the piece of information (*I*) is made of two independent pieces of information, $I_{1}$ and $I_{2}$, in the sense that $l\left(\omega ,I\right)=l\left(\omega ,I_{1}\right)+l\left(\omega ,I_{2}\right)$, for every ω in Ω, then the posterior probability satisfies the following:
  (5) $$\psi \left(l\left(\cdot ,I_{2}\right),\psi \left(l\left(\cdot ,I_{1}\right),p\left(\cdot \right)\right)\right)\equiv \psi \left(l\left(\cdot ,I_{1}\right)+l\left(\cdot ,I_{2}\right),p\left(\cdot \right)\right).$$
  This ensures we end up with $p_{I_{1},I_{2}}\left(\omega \right)$ as the same object whether we update with $\left(I_{1},I_{2}\right)$ together or $\left\{I_{1},I_{2}\right\}$ one after the other.
3. If l(ω,I)=∞ for every $\omega \in B^{c}$ and some B⊂Ω, then
  (6) $$\psi \left(l\left(\cdot ,I\right),p\left(\cdot \right)\right)\left(\omega \right)=\left\{\begin{array}{cc} \psi (l\left(\cdot ,I\right){|}_{B},p_{J(B)}\left(\cdot \right))\left(\omega \right) & \mathrm{if}\;\omega \in B\\ 0 & \mathrm{if}\;\omega \notin B, \end{array}\right.$$
  where ${l\left(\cdot ,I\right)|}_{B}$ is the restriction of l(·,I) to *B*, J(B) is the information that *B* certainly occurs or has occured, and $p_{J(B)}$ is *p* restricted and normalized to *B*, i.e., $p_{J(B)}\left(\omega \right)=p\left(\omega \right)1\left(\omega \in B\right)/\sum_{\omega \in B}p\left(\omega \right)$. In other words, outcomes with infinite loss are disregarded in the updating.
4. Lower evidence (larger loss) for a state should yield smaller posterior probabilities under the same prior. So, if for some A⊂Ω, $l\left(\omega ,I_{1}\right)>l\left(\omega ,I_{2}\right)$ for ω∈A⊂Ω and $l\left(\omega ,I_{1}\right)=l\left(\omega ,I_{2}\right)<\infty$ for $\omega \in A^{c}$, then
  $$\sum_{\omega \in A}\psi \left(l\left(\cdot ,I_{1}\right),p\left(\cdot \right)\right)\left(\omega \right)<\sum_{\omega \in A}\psi \left(l\left(\cdot ,I_{2}\right),p\left(\cdot \right)\right)\left(\omega \right).$$
5. If l(ω,I)≡constant, then ψ(l(·,I),p(·))≡p(·). That is, if the observation provides no information about ω, since the loss function is a constant, then the posterior is the same as the prior.

As a consequence of these, we have the following theorem:

**Theorem** **1.** *For |Ω|<∞, with axioms 1 to 5, it is uniquely implied that*

(7) $$p_{I}\left(\omega \right)=\frac{\exp\{-\lambda l(\omega ,I)\}p(\omega )}{\sum_{\omega^{\prime }\in \Omega }\exp\left\{-\lambda l\left(\omega^{\prime },I\right)\right\}p\left(\omega^{\prime }\right)},$$

*for some λ>0*.

In order to prove Theorem 1, the following Lemma is useful.

**Lemma** **1.** *Let |Ω|<∞. Axioms 1 to 5 imply:*

*6*. *If $\tilde{l}\left(\cdot ,I\right)\equiv l\left(\cdot ,I\right)+c$ for some constant c, then $\psi \left(\tilde{l}\left(\cdot ,I\right),p\left(\cdot \right)\right)\equiv \psi \left(l\left(\cdot ,I\right),p\left(\cdot \right)\right)$*.
*7*. *for any set B⊂Ω*,
  (8) $$\frac{\psi (l(\cdot ,I),p(\cdot ))(\omega )}{\sum_{\omega^{\prime }\in B}\psi \left(l\left(\cdot ,I\right),p\left(\cdot \right)\right)\left(\omega^{\prime }\right)}=\psi \left(l\left(\cdot ,I\right){|}_{B},p_{J(B)}\left(\cdot \right)\right)\left(\omega \right),$$
  *for every ω in B. This means that whether we update the prior probability p(·) restricted to set B, or update the prior probability p(·) and then restrict to set B, we obtain the same update*.

**Proof of** **Lemma 1.** Combination of axioms 2 and 5 yield axiom 6. Indeed, one just needs to consider (5) the case where the loss function $l(\cdot ,I_{1})$ or $l(\cdot ,I_{2})$ is constant.

Combining conditions 3 and 5, one obtains that updating with the loss (1) corresponding to information J(B) yields the conditional probability $p_{J(B)}\left(\omega \right)=p\left(\omega \right)I_{B}\left(\omega \right)/P\left(B\right)$ that is obtained by restricting and normalizing the prior to *B*. Now, consider $I_{1}=J\left(B\right)$ and let $I_{2}$ be any piece of information associated with a given loss function (*l*). The sum of the two corresponding losses is infinite on $B^{c}$ and coincides with *l* on *B*, which is the loss considered in condition 3. Therefore, based on condition 2, condition 7 is obtained. ☐

**Proof of** **Theorem 1.** In the two points case, say $\Omega =\{\omega_{0},\omega_{1}\}$, the prior probability (p(·)) is determined by a real number (*z*) in the unit interval (i.e., $z=p(\omega_{0})$), and the loss l(ω,I) takes only two values, say $l_{0}$ and $l_{1}$ (i.e., $l\left(\omega_{0},I\right)=l_{0}$, $l\left(\omega_{1},I\right)=l_{1})$. According to condition 6, we can replace the pair $\left(l_{1},l_{0}\right)$ with $\left(l_{1}-l_{0},0\right)$. Therefore, the posterior probability $p_{I}\left(\cdot \right)$ is a function of $l=l_{1}-l_{0}$ and *z*, say $\bar{\psi }\left(l,z\right)$. In order to proceed, it is convenient to think in terms of odds rather than probabilities. So, if $z=p(\omega_{0})$, we can consider t=z/(1−z) (i.e., z=t/(1+t)). We can do the same with the posterior ($p_{I}\left(\omega_{0}\right)$). The posterior odds, $p_{I}\left(\omega_{0}\right)/\left\{1-p_{I}\left(\omega_{0}\right)\right\}$, are a function of the loss difference (*l*) and the prior odds (*t*), which we denote by ϕ(l,t).

To deal with the odds, (5) becomes

(9) ϕ(l+h,t)=ϕ(l,ϕ(h,t)),

where *h* is a replication of *l* with a possible different *I*. Moreover, with a constant loss, the posterior is equal to the prior (condition 5), i.e.,

(10) ϕ(0,t)=t,

for every t>0.

At this stage, we consider a prior with three mass points, say $\Omega =\{\omega_{1},\omega_{2},\omega_{3}\}$. The prior is given by $\left\{z_{1},z_{2},1-z_{1}-z_{2}\right\}$ (i.e., $p\left(\omega_{1}\right)=z_{1}$ and $p\left(\omega_{2}\right)=z_{2}$), with $l\left(\omega_{i},I\right)=l_{i}$, for i=1,2,3. The loss can be zero at one point without loss of generality (condition 6), so it takes values into the set $\left\{l_{1},l_{2},0\right\}$. Let us consider the updating rule, ϕ, for priors with just two mass points. We can use this to update the conditional probability of $\left\{\omega_{1}\right\}$ given $\left\{\omega_{1},\omega_{3}\right\}$, i.e., $z_{1}/\left(z_{1}+z_{3}\right)$. To this aim, we update the prior with masses $z_{1}/\left(z_{1}+z_{3}\right)$ and $z_{3}/\left(z_{1}+z_{3}\right)$ considering just the loss values, $\left(l_{1},0\right)$, i.e., disregarding the point $\omega_{2}$ with its loss ($l_{2}$). In other words, we aim to obtain the right-hand-side of (8) and apply condition 7 given by Lemma 1. $t_{1,3}$ denotes the odds corresponding to the conditional probability of $\omega_{1}$ given $\left\{\omega_{1},\omega_{3}\right\}$, that is $t_{1,3}=z_{1}/z_{3}$. $t_{1,3}$ is updated on the basis of the loss values ($l_{1}$) and zero, i.e., with $\phi (l_{1},t_{1,3})$. Similarly, we define $t_{1,2}=z_{1}/z_{2}$ and $t_{2,3}=z_{2}/z_{3}$. We update $t_{1,2}$ on the basis of $l_{1}$ and $l_{2}$, i.e., with $\phi (l_{1}-l_{2},t_{1,2})$ and $t_{2,3}$ with $\phi (l_{2},t_{2,3})$. Clearly, $t_{1,3}=t_{1,2}t_{2,3}$, and this factorization of conditional odds has to hold also after updating, i.e.,

(11) $$\phi \left(l_{1},t_{1,3}\right)=\phi \left(l_{1}-l_{2},t_{1,2}\right)\phi \left(l_{2},t_{2,3}\right),$$

where $t_{1,3}=t_{1,2}\cdot t_{2,3}$. Formally, this identity is a consequence of (8), i.e., updating the conditional probability is the same as conditioning the updated probability. Since (11) must hold for every $t_{2,3},t_{1,2}>0$, and for every $l_{1},l_{2}\in R$,

$$\phi \left(l_{1},ts\right)=\phi \left(l_{1}-l_{2},t\right)\phi \left(l_{2},s\right),$$

for every t,s>0 and $l_{1},l_{2}\in R$. If $l_{2}=0$, and say $l_{1}=l$, recalling (10),

ϕ(l,ts)=ϕ(l,t)s,

for every t,s>0 and every real *l*. Letting t=1, we find that

(12) ϕ(l,s)=ϕ(l,1)s,

for every s>0 and every l∈R. A combination of (9) and (12) yields ϕ(l+h,1)=ϕ(l,ϕ(h,1))=ϕ(l,1)ϕ(h,1) for every h,l∈R which, in turn, implies by monotonicity that ϕ(l,1)=exp(−λl) for some λ∈R. Indeed, according to condition 4, ϕ(l,1) is a monotone, not an increasing function of *l*. This implies that λ must be positive. Hence,

(13) ϕ(l,t)=exp(−λl)t,

for every l∈R and every t>0. In this way, we are basically done with the two points case.

Let us now consider the general finite case. We want to update the prior p(·) with mass points at $\left\{\omega_{1},\ldots ,\omega_{m}\right\}$ given by $\left\{z_{1},\ldots ,z_{m-1},1-\left(z_{1}+\ldots ,z_{m-1}\right)\right\}$, where $z_{1},\ldots ,z_{m-1}$ are non-negative and their sum is less than or equal to one, and it is convenient to set $z_{m}:=1-\left(z_{1}+\cdots +z_{m-1}\right)$. In terms of odds, the prior is given by the vector $\left(t_{1},\ldots ,t_{m-1}\right)$ (being $t_{i}=z_{i}/\left(1-z_{i}\right)$, or equivalently, $z_{i}=t_{i}/\left(1+t_{i}\right)=1-1/\left(1+t_{i}\right)$, i=1,…,m−1), where $t_{i}\geq 0$ and $t_{1}/\left(1+t_{1}\right)+\cdots +t_{m-1}/\left(1+t_{m-1}\right)\leq 1$. Here, it is convenient to set $t_{m}:={\left\{{\left\{1-t_{1}/\left(1+t_{1}\right)+\cdots +t_{m-1}/\left(1+t_{m-1}\right)\right\}}^{-1}-1\right\}}^{-1}$. Moreover, we consider an $R^{m-1}$ valued function $\phi \left(l,t\right)=(\phi_{1}\left(l,t\right),\ldots ,\phi_{m-1}\left(l,t\right)),$ which provides the vector of the updated odds as being $l=(l_{1},\ldots ,l_{m})$ and $t=(t_{1},\ldots ,t_{m-1})$. Now the question is how we could recover ϕ from ϕ, where the latter gives the updating rule for the two points case. Recall the notation used for the conditional odds, i.e., $t_{i,j}=z_{i}/z_{j}$, is the odds corresponding to the conditional probability of $\left\{\omega_{i}\right\}$ given $\left\{\omega_{i},\omega_{j}\right\}$ for a distinct i,j=1,…,m. We can see that $t_{j}=z_{j}/\sum_{i\neq j}z_{i}={\left(\sum_{i\neq j}t_{i,j}\right)}^{-1}$. According to (8), this identity will also have to be satisfied by the updated odds. Since we update $t_{i,j}$ with $\phi (l_{i}-l_{j},t_{i,j})$, we must have

$$\phi_{j}\left(l_{1},\ldots ,l_{m};t_{1},\ldots ,t_{m-1}\right)={\left(\sum_{i\neq j}\phi \left(l_{i}-l_{j},t_{i,j}\right)\right)}^{-1},$$

which by (13) becomes:

(14) $$\phi_{j}\left(l_{1},\ldots ,l_{m};t_{1},\ldots ,t_{m-1}\right)=\exp\left(-\lambda l_{j}\right){\left(\sum_{i\neq j}\exp\left(-wl_{i}\right)t_{i,j}\right)}^{-1}.$$

$t_{i,j}=z_{i}/z_{j}$, (14) becomes

$$\phi_{j}\left(l_{1},\ldots ,l_{m};t_{1},\ldots ,t_{m-1}\right)=\frac{\exp\left(-\lambda l_{j}\right)z_{j}}{\sum_{i\neq j}\exp\left(-\lambda l_{i}\right)z_{i}},$$

and the updated probability of $\left\{\omega_{j}\right\}$ is

$$1-{\left(1+\phi_{j}\left(l_{1},\ldots ,l_{m};t_{1},\ldots ,t_{m-1}\right)\right)}^{-1}=\frac{\exp\left(-\lambda l_{j}\right)z_{j}}{\sum_{i=1}^{m}\exp\left(-\lambda l_{i}\right)z_{i}}.$$

In this way, we have shown how to extend our coherent updating rule from the two points case to the general finite case.  ☐

The proof implies the existence of λ>0, and this is no surprise since loss functions are only defined up to scalar factors. Hence, the setting of λ is a calibration issue and to set this, we need more information that is not associated with any specific information (*I*). So, l(ω) is set to be the loss function that contains initial beliefs at the outset before any further information (*I*) is obtained, and this l(ω) is defined to be on the same scale as any l(ω,I). We regard $l\left(\omega \right)=l\left(\omega ,I_{0}\right)$, where $I_{0}$ is the prior information used to set *p*.

The prior expected loss is then defined by

$$L=\lambda \sum_{\omega \in \Omega }l\left(\omega \right)p\left(\omega \right).$$

Now, entropy is also regarded as an expected loss based on the self-information loss function −logp(ω). Hence, we also have expected loss/entropy which is measured as

$$E=-\sum_{\omega \in \Omega }p\left(\omega \right)\log p\left(\omega \right),$$

and so calibration is achieved by matching these expected losses, so that

$$\lambda =-\frac{\sum_{\omega \in \Omega }p\left(\omega \right)\log p\left(\omega \right)}{\sum_{\omega \in \Omega }l\left(\omega \right)p\left(\omega \right)}.$$

Finally, in this section, we note that it is quite straightfoward to extend the uniqueness argument to all countably infinite Ω, which replaces the uniqueness argument for all Ω. However, we need more work to extend separate uniqueness to the general Ω.

### 2.2. An Illustration

The loss function *l* is chosen by the decision-maker on the basis of the available information. Such information sometimes happens to be stochastic, i.e., belonging to a set *B* of outcomes to which a probability is assigned. If this is the case, one should update the probability by means of the usual conditional probability. It is tantamount to use the loss function (1) in (3). If the available information is not stochastic, then one can resort to the approach described in the present paper to properly assess the loss function *l*. A simple and very concrete example is now presented. Consider a horse race in which six horses participate. In order to decide how to bet, one assesses the probability for each horse to win. p(j) demotes the probability that horse number *j* wins for j∈{1,…,6}. In this example, the elements of Ω are the numbers corresponding to the horses participating in the race, of which one will win.

Before the race begins, it starts raining. Since conditions have changed, the probabilities need to be updated. It is problematic to pursue this aim by resorting to the current definition of conditional probability. In fact, this requires knowledge of the probability that it rains and that the horse number *j* wins. As an alternative, one could calculate the conditional probabilities of a win for each horse by applying Bayes’ theorem, which requires the probability that it rains given the win of horse *j*. However, it is raining and the race has not yet been run!

It is therefore appropriate to resort to the definition of a conditional probability distribution given in this paper. So, p(j) is the prior probabilty that horse *j* wins and I= is the probability that “it is raining”. l(j) be the loss function, i.e., the loss incurred to the bookmaker, that is, the governor of the probabilities, if horse *j* wins. To elaborate, such an l(j) could be the assessed loss if the bookmaker puts all the takings received or a fixed amount on horse *j* to win.

The bookmaker then, on the same scale, assesses l(j,I) for each *j* which adjusts the loss l(j). Obviously, a higher score will be given to those horses whose ability to run is more affected by the rain. In this way, one can use the ideas for setting λ to get $\tilde{l}\left(j\right)=\lambda l\left(j,I\right)$. The updated probability that the *j*-th horse wins is revised to

$$p_{I}\left(j\right)=\frac{\exp\{-\tilde{l}\left(j\right)p\left(j\right)\}}{\sum_{i=1}^{6}\exp\left\{-\tilde{l}\left(i\right)p\left(i\right)\right\}},$$

for j=1,…,6.

## 3. Discussion

We have established a framework in which we can update probabilities in the light of general, i.e., non-stochastic, information. Given that we cannot connect the information and the outcome of interest via a probability model, we do so through a loss function. Minimizing a cumulative loss function involving the information on one side and the probability distribution on the other yields the updated probability distribution. When the information is stochastic, we employ the self information loss function; the solution then reverts to the standard definition of conditional probability.

We believe the framework has direct implications for Bayesian inference where “word of mouth” information from experts is often obtained. It is interesting to note that our framework allows this information to update *p* at any point. Usually, the non-stochastic information *I* comes before the stochastic information given by an event *B* included in the probability framework, but in our approach, we can have *B* before *I*.
